# Emergence of Ceftazidime‐Avibactam‐Induced KPC Variants (KPC‐25/127) in Intracranial Infection and Implications for Clinical Management

**DOI:** 10.1002/mbo3.70363

**Published:** 2026-07-01

**Authors:** Ke Lei, Ying Tian, Chaoliang Xiong, Jing Lei, Dong Chen, Xiangni Bai, Mohd H. Abdul‐Aziz, Jiao Xie, Zeshi Liu

**Affiliations:** ^1^ Department of Clinical Laboratory The Second Affiliated Hospital of Xi'an Jiaotong University Xi'an China; ^2^ Department of Clinical Laboratory XD Group Hospital Xi'an China; ^3^ University of Queensland Centre for Clinical Research (UQCCR), Faculty of Medicine The University of Queensland Brisbane QLD Australia; ^4^ Department of Pharmacy The Second Affiliated Hospital of Xi'an Jiaotong University Xi'an China

**Keywords:** ceftazidime‐avibactam, Klebsiella pneumoniae, KPC‐127, KPC‐25

## Abstract

The emergence of novel *Klebsiella pneumoniae* carbapenemase (KPC) variants contributing to clinical treatment failure poses a substantial threat to public health. To inform the clinical management of infections caused by KPC‐variant‐harboring *K. pneumoniae*, this study reports the complex evolutionary trajectory of ceftazidime‐avibactam (CAZ‐AVI)‐resistant KPC‐variant‐harboring *K. pneumoniae* in a single patient during CAZ‐AVI treatment. Three KPC‐producing *K. pneumoniae* (KPC‐KP) isolates were obtained from a patient with *K. pneumoniae* infection who received sequential treatment with imipenem followed by CAZ‐AVI. Isolate K2, harboring *bla*_KPC‐2_, exhibited carbapenem resistance. After 12 days of CAZ‐AVI administration, isolate K25 (carrying *bla*_KPC‐25_) showed CAZ‐AVI resistance but remained susceptible to imipenem. In contrast, isolate K127 (harboring *bla*_KPC‐127_ firstly identified by our group), recovered after combined CAZ‐AVI and imipenem therapy, displayed dual resistance to both CAZ‐AVI and carbapenems; this carbapenem resistance was attributed to outer membrane protein mutations combined with *bla*_KPC‐127_ overexpression. Analysis of the genetic regions revealed that all subtypes were located within a conserved genetic background: IS*26*‐IS*Kpn8*‐*bla*_KPC_‐ΔIS*Kpn6*‐Δ*tnpR*‐Tn*1721*. Cloning and heterologous expression of the KPC variants, followed by enzymatic kinetic analyses and minimum inhibitory concentration (MIC) determinations, confirmed that the variants altered the MICs of CAZ‐AVI and carbapenems. Compared with the KPC‐2 protein, both variant proteins exhibited reduced hydrolytic activity against ceftazidime. Additionally, the half‐maximal inhibitory concentration (IC_50_) of avibactam against the KPC variants was significantly higher than that of the wild‐type KPC‐2 protein. These results indicate that the KPC variant proteins have low affinity for avibactam, thereby reducing susceptibility to this inhibitor.

## Graphical abstract

Novel KPC‐25 and first‐reported KPC‐127 sequentially evolved in one patient under CAZ‐AVI pressure, distinct mutations remodel enzyme activity to drive differential multidrug resistance.

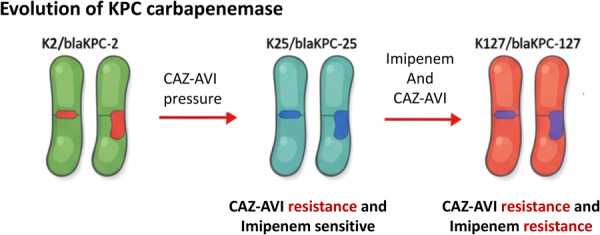

## 1 Introduction

Global dissemination of carbapenem‐resistant *Klebsiella pneumoniae* (CRKP) across healthcare settings worldwide constitutes a significant challenge to clinical anti‐infective treatment (Di Pilato et al. 2024). Production of carbapenemases, with *Klebsiella pneumoniae* carbapenemase (KPC) being the predominant type, serves as the primary determinant underlying CRKP resistance to carbapenems (Chen et al. 2014; da Silva et al. 2012; Munoz‐Price et al. 2013). Presently, novel β‐lactam/β‐lactamase inhibitor (BL/BLI) combinations—such as ceftazidime‐avibactam (Shirley 2018), meropenem‐vaborbactam (Novelli et al. 2020), and imipenem‐relebactam (Zhanel et al. 2018)—are regarded as the first‐line effective therapeutic strategies for infections caused by carbapenemase‐producing *K. pneumoniae* (KPC‐KP).

Given its strong antibacterial activity against KPC‐KP, ceftazidime‐avibactam is clinically used for complex intra‐abdominal infections (cIAI), hospital‐acquired pneumonia (HAP), and ventilator‐associated pneumonia (VAP) caused by KPC‐KP (Carmeli et al. 2016). However, since FDA approval in 2015, KPC‐KP strains have evolved from susceptible to ceftazidime‐avibactam‐resistant during treatment, especially those harboring novel KPC variants derived from prototype KPC‐2 or KPC‐3 (Hobson et al. 2022; Shen et al. 2022, 2025). To date, the NCBI database (https://www.ncbi.nlm.nih.gov/pathogens/refgene/#gene_family:(blaKPC)) has recorded over 250 KPC variant types globally.

Evidence indicates that KPC variants arise from amino acid mutations (substitutions, insertions, deletions, tandem repeats), which alter KPC enzyme structure, modifying its substrate spectrum and inhibitor affinity (Birgy et al. 2024). Nevertheless, there are no international consensus guidelines for treating KPC variant‐harboring strain infections. Sporadic studies suggest salvage therapies such as meropenem‐avibactam or ceftazidime‐avibactam combined with imipenem, but supporting clinical evidence is limited (Ding et al. 2022). Notably, ceftazidime‐avibactam resistance in KPC variant strains may be associated with restored carbapenem susceptibility, particularly to imipenem, making imipenem a preferred choice for such infections (Ding et al. 2023). However, imipenem‐susceptible mutant KPC‐KP can revert to carbapenem‐resistant strain under antimicrobial pressure. Additionally, coexistence of mutant and non‐mutant populations is possible, with carbapenem treatment selecting for KPC‐2‐harboring strains (Tang et al. 2024).

Repeated *bla*_KPC_ mutations under different antibiotic pressures complicate patient management and challenge clinical therapy. This study reports the complex evolution of ceftazidime‐avibactam‐resistant KPC variants in a single patient during sequential imipenem and ceftazidime‐avibactam therapy. Three clinical KPC‐KP strains were isolated: prototype *bla*_KPC‐2_, variants *bla*_KPC‐25_, and *bla*_KPC‐127_ (first reported CRKP producing KPC‐127). Accurate KPC variant identification, combined with in vitro susceptibility testing, carbapenemase phenotype detection, and whole‐genome sequencing, is crucial for timely antibiotic adjustment and improved clinical outcomes.

## 2 Methods

### 2.1 Species Identification and Antimicrobial Susceptibility Testing

Strain identification was performed by MALDI‐TOF MS (bioMérieux, France). The minimal inhibitory concentration (MIC) of antimicrobials was determined by the broth microdilution method recommended by the Clinical and Laboratory Standards Institute (CLSI) using ATCC 25922 and ATCC 27853 as quality control. Quality control and MIC results were interpreted according to the CLSI breakpoints (Humphries et al. 2021) for all agents except tigecycline (S, ≤ 0.5 mg/L; R, > 0.5 mg/L), which were interpreted according to EUCAST and US FDA breakpoints, respectively (Wagoner et al. 2021; Giske et al. 2022). The NG ‐Test CARBA5 (FOSUN DIAGNOSTICS) and the carbapenemase inhibitor enhancement test were used for rapid detection of the carbapenemase (Routsias et al. 2020).

### 2.2 PCR, Cloning Experiments, and DNA Sequencing

Genomic DNA was isolated from K. pneumoniae strains producing KPC‐2, KPC‐25, and KPC‐127 using the DNA Mini Kit (Vazyme, China). The extracted genomic DNA served as templates for the amplification of *bla*_KPC‐2_‐like genes via polymerase chain reaction (PCR). The PCR assay was performed with the specific primers KPC‐CF (5‘‐CCATGATTACGAATTGTGCGCGGAACCCCTATTTG‐3') and KPC‐CR (5‘‐CGACTCTAGAGGATCCAATAGATGATTTTCAGAGCCTTAC‐3'), which are capable of amplifying *bla*_KPC‐2_‐like gene sequences. Subsequently, the obtained PCR amplicons were cloned into the pHSG398 plasmid, positioned downstream of the pLac promoter and in the same orientation required for phenotypic analyses. All recombinant plasmids were sequenced using M13F promoter and M13R terminator primers prior to transformation into E. coli BL21 competent cells. For recombinant protein expression, the coding sequences of KPC‐2, KPC‐25, and KPC‐127 excluding the signal peptide (The first 30 amino acids) were amplified by PCR using primers KPC‐28a‐F (5'‐AATGGGTCGCGGATCCGCGGAACCATTCGCTAAACTCG‐3') and KPC−28a‐R (5'‐GACGGAGCTCGAATTTTACTGCCCGTTGACGCCC‐3'). These amplified fragments were inserted into the pET28a expression vector. Post‐transformation, the recombinant plasmids were re‐sequenced with T7 promoter and T7 terminator primers to verify sequence accuracy. Nucleotide sequence analyses were conducted using bioinformatics tools available on the National Center for Biotechnology Information (NCBI) website (http://www.ncbi.nlm.nih.gov).

### 2.3 Whole Genome Sequencing and Bioinformatics Analysis

Genomic DNA was isolated from the test isolates using a commercial Qiagen kit. This genomic DNA was first subjected to sequencing on the Illumina MiSeq platform (Illumina Inc.) with a paired‐end strategy (2×300 bp) and additionally sequenced using Oxford Nanopore Technologies (ONT) platforms. Short reads from Illumina and long reads from ONT underwent de novo hybrid assembly using Unicycler v0.4.8. Genome annotation, as well as the prediction of multilocus sequence typing (MLST), plasmid replicons, and resistance genes, were accomplished with the following bioinformatics tools: RAST version 2.0 (https://rast.nmpdr.org/rast.cgi), MLST (Matsumura 2013), PlasmidFinder (Carattoli et al. 2014), and Resfinder (Florensa et al. 2022). Single nucleotide polymorphism (SNP) analysis was performed via BacWGSTdb (http://bacdb.cn/BacWGSTdb/Tools.php), with *K. pneumoniae* HS11286 serving as the reference strain. Plasmid draft maps were generated using Proksee (https://proksee.ca/), while the genetic environment of the blaKPC gene was characterized using Chiplot (https://www.chiplot.online/). All statistical analyses were conducted with Prism 10 software.

### 2.4 Protein Purification

overnight cultures of E. coli BL21 harboring recombinant pET28a‐KPC plasmids were used to inoculate 2 L of LB broth supplemented with 50 mg/L kanamycin. Bacteria were cultured at 37°C until the optical density at 600 nm (OD_600_) reached 0.6. Expression of β‐lactamase genes was induced overnight at 22°C with 1 mM isopropyl β‐D‐1‐thiogalactopyranoside (IPTG). Bacterial cultures were centrifuged at 6000 × g for 15 min, and the resulting pellets were resuspended in binding buffer (10 mM imidazole, 25 mM sodium phosphate, pH 7.4, and 300 mM NaCl). Bacterial cells were disrupted by sonication, and cellular debris was removed via two sequential centrifugation steps (10,000 × g for 1 h at 4°C). The supernatant was further centrifuged at 96,000 × g for 1 h at 4°C. The soluble fraction was filtered and loaded onto a HisTrap HP column (Yeasen), and target proteins were eluted with elution buffer (500 mM imidazole, 25 mM sodium phosphate, pH 7.4, and 300 mM NaCl). Subsequent gel filtration was performed using a Superdex 75 column (GE Healthcare) equilibrated with 100 mM sodium phosphate buffer (pH 7.0) containing 150 mM NaCl. Protein purity was evaluated by sodium dodecyl sulfate‐polyacrylamide gel electrophoresis (SDS‐PAGE). Pooled target protein fractions were dialyzed against 10 mM Tris‐HCl (pH 7.6) and concentrated using Vivaspin columns. Protein concentrations were determined by measuring OD_280_ values, with extinction coefficients calculated using the ProtParam tool (Swiss Institute of Bioinformatics online resource portal).

### 2.5 Steady‐State Kinetic Parameters

Enzymatic kinetic parameters were determined using the purified KPC‐2, KPC‐25, and KPC‐127 β‐lactamases in 100 mM sodium phosphate buffer (pH 7.0). All kinetic reactions were performed under pseudo‐first‐order initial rate conditions with a large excess of substrate relative to enzyme, which is the standard condition for β‐lactamase kinetic analysis. The initial hydrolysis rates of β‐lactam substrates were measured using a UV‐2700 spectrophotometer (Shimadzu, Japan). Saturation was achieved for ceftazidime, imipenem and meropenem, enabling reliable calculation of individual *Kcat* and *Km* values shown in Table 3. The 50% inhibitory concentration (IC_50_) of avibactam against KPC enzymes was determined using nitrocefin as the reporter substrate. Briefly, the enzyme was mixed with avibactam at concentrations ranging from 0 to 20 μM in phosphate‐buffered saline (PBS) and incubated for 15 min. Nitrocefin was then added to a final concentration of 100 μM, and the hydrolysis rate of nitrocefin was monitored at 482 nm for 30 min. IC_50_ values were calculated using the dose‐response inhibition equation with a variable slope (four parameters) in Prism software.

## 3 Results

### 3.1 Case Information

**Figure 1:**
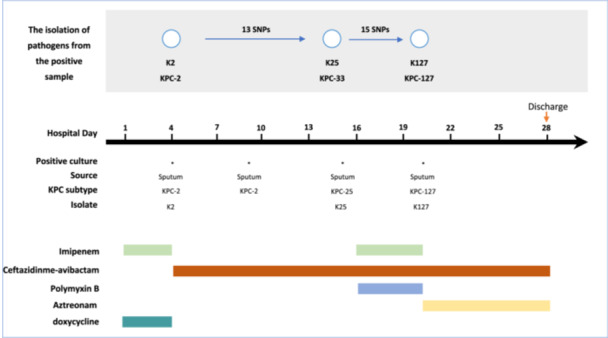
Timeline clinical history of the case patient.

Three strains of *K. pneumoniae* (designated as K2, K25, and K127) were isolated from a 45‐year‐old female patient diagnosed with intracranial infection upon admission to **The Second Affiliated Hospital of Xi'an Jiaotong University**. Empirical antimicrobial therapy was initiated with doxycycline (0.1 g, q12h) and imipenem (0.5 g, Q6h). The first *K. pneumoniae* strain (K2) harboring the *bla*_KPC‐2_ gene was recovered from sputum prior to ceftazidime‐avibactam (CAZ‐AVI) treatment. In vitro antimicrobial susceptibility testing (AST) demonstrated that strain K2 was resistant to carbapenems; therefore, the antimicrobial regimen was adjusted from imipenem plus doxycycline to ceftazidime‐avibactam (2.5 g, Q12h) for an 11‐day course. Although the patient's body temperature stabilized during CAZ‐AVI treatment, infection markers failed to return to normal ranges, and a CAZ‐AVI‐resistant *K. pneumoniae* strain (K25) was subsequently isolated. The therapeutic strategy was then modified to a combination of meropenem (1 g, q8h) and ceftazidime‐avibactam (2.5 g, Q12h). During this combined therapy period, a *K. pneumoniae* strain (K127) exhibiting dual resistance to both meropenem and ceftazidime‐avibactam was isolated from a sputum specimen, with a time interval of no more than 5 days from the isolation of K25. Due to the poor clinical response to the combined meropenem‐CAZ‐AVI regimen, the therapeutic scheme was further adjusted to ceftazidime‐avibactam (2.5 g, q8h) in combination with aztreonam (0.5 g, q8h). Fortunately, the patient achieved clinical recovery following this regimen adjustment. A comprehensive timeline of the patient's clinical course and antimicrobial treatment history is presented in Figure 1.

### 3.2 Antimicrobial Susceptibility Profiles of *K. pneumoniae* Clinical Isolate, Transformant, and Recipient to Antimicrobial Agents

The antimicrobial susceptibility profiles of the clinical *K. pneumoniae* strains (K2, K25, K127), *bla*_KPC_‐positive transconjugants, transformants, and the recipient strain are summarized in Table 1. Cloning experiments confirmed that the presence of KPC variants mediated significant alterations in the minimum inhibitory concentrations (MICs) of ceftazidime‐avibactam, imipenem, and meropenem. Compared with the recipient strain carrying the empty vector, strains expressing KPC‐25 and KPC‐127 exhibited 64‐fold and 256‐fold increases in ceftazidime‐avibactam MICs, respectively. Notably, KPC‐25 expression did not affect the MICs of carbapenems (imipenem and meropenem), whereas KPC‐127 expression resulted in a fourfold increase in imipenem MIC and a twofold increase in meropenem MIC. The carbapenem hydrolysis capacities differed among the KPC subtypes: KPC‐127 displayed partial loss of carbapenem hydrolytic activity, while KPC‐25 showed complete abrogation of this activity (Table 1).

**Table 1:** Antimicrobial susceptibility profiles of clinical strains of K. pneumoniae (K2, K25, and K127), blaKPC‐positive conjugants, transformants and recipient strain.

Antimicrobials	MIC (μg/mL)						
	K2 (KPC‐2)	K25 (KPC‐25)	K127 (KPC‐127)	pHSG398‐*E. coli* DH5a	KPC‐2‐*E. coli* DH5a	KPC‐25‐*E. coli* DH5a 167_168dupLE	KPC‐127‐*E. coli* DH5a 167_168dupLE 172 A > T
Imipenem	128	0.25	8	0.125	32	0.125	0.5
Meropenem	128	2	16	<=0.06	16	<= 0.06	1
Ceftazidime	> 128	> 128	> 128	1	32	32	64
Ceftazidime‐avibactam	8	> 64	> 64	0.125	0.125	32	> 64
Imipenem‐relebactam	0.5	0.5	0.5	0.125	0.25	0.125	0.125
Aztreonam‐avibactam	2	1	1	0.25	0.25	0.125	0.25
Imipenem‐relebactam	0.5	0.5	0.5	0.125	0.25	0.125	0.125
Meropenem‐vaborbactam	1	0.5	0.5	<=0.06	<=0.06	<=0.06	<=0.06
Aztreonam	> 128	> 128	> 128	0.125	128	2	1
Amikacin	> 128	> 128	> 128	0.25	0.25	0.5	1
Ciprofloxacin	> 8	> 8	> 8	<= 0.06	<= 0.06	<= 0.06	<= 0.06
Polymyxin B	8	0.25	0.25	0.25	0.25	0.5	0.5
Tigecycline	0.5	0.25	0.5	0.25	0.25	0.25	0.25

### 3.3 Molecular Analysis of *K. pneumoniae* Isolates Harboring bla_KPC_ Variants

**Figure 2:**
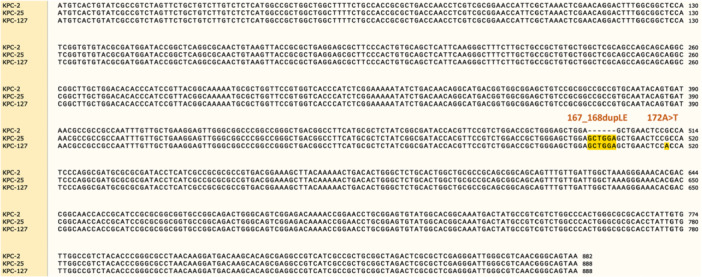
Amino acid sequence alignment of KPC‐2, KPC‐25 and KPC‐127 using snapgene. Amino acids that match the reference are marked with yellow highlighting.

Whole‐genome sequencing (WGS) analysis revealed that *K. pneumoniae* strains K2, K25, and K127 all belonged to sequence type 11 (ST11). PCR amplification and sequencing confirmed that strain K2 carried bla_KPC‐2_, while strains K25 and K127 harbored bla_KPC‐25_ and bla_KPC‐127_, respectively. Amino acid sequence alignment showed that bla_KPC‐25_ differs from bla_KPC‐2_ by a 167_168dupLE insertion in the Ω‐loop region, whereas bla_KPC‐127_ contains both the 167_168dupLE insertion and a 172 A > T point mutation. Single‐nucleotide polymorphism (SNP) analysis identified 13 SNPs between the KPC‐25‐harboring strain and the KPC‐2‐harboring strain, and 19 SNPs between the KPC‐127‐harboring strain and the KPC‐2‐harboring strain, indicating a high degree of genetic homology among these three clinical isolates. The amino acid sequence alignment of the novel bla_KPC_ allelic variants relative to bla_KPC‐2_ is shown in Figure 2.

**Figure 3:**
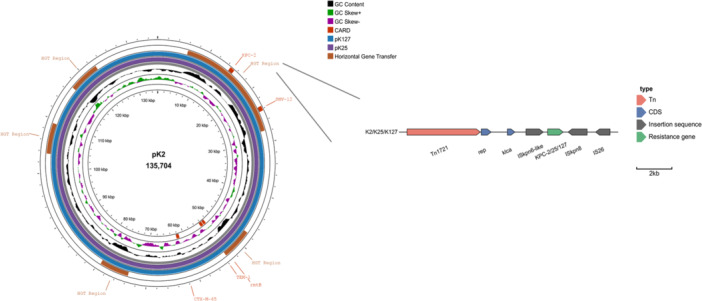
The plasmid structure carrying bla_KPC_. Different colors represent relevant information such as drug resistance genes and horizontal transfer genes.

Third‐generation sequencing further demonstrated that all three strains carried the chromosomal aadA2 and bla_SHV‐182_ genes, as well as plasmid‐borne resistance genes including bla_CTX‐M‐65_, bla_KPC_ (variant‐specific alleles), bla_SHV‐12_, and bla_TEM‐1B_ (Figure 3). Porin gene analysis revealed mutations in the OmpK35 gene in all three strains; these mutations were identified as insertions leading to frameshift mutations, which resulted in the production of non‐functional OmpK35 proteins. In contrast, the ^Omp^K36 gene retained the wild‐type sequence in strains K2 and K25, while a GD insertion was detected in the ^Omp^ *K36* gene of strain K127. All three strains carried the wild‐type penicillin‐binding protein 3 (PBP3) encoded by the ftsI gene, and none harbored *bla*_PER_ or AmpC β‐lactamase genes—resistance determinants previously associated with ceftazidime‐avibactam resistance.

Analysis of the genetic environments flanking bla_KPC‐2_ and the bla_KPC_ variants showed that all bla_KPC_ alleles were located within the conserved genetic context IS26‐ISKpn8‐bla_KPC_‐ΔISKpn6‐ΔtnpR‐Tn1721 (Figure 3). BLAST homology analysis revealed that numerous *bla*_KPC‐2_ variants have been identified within Tn1721‐associated transposons, with a predominant distribution in clinical isolates from China. Using the *bla*_KPC‐2_‐positive plasmid pP10159‐3 as a reference, the transposon structure was confirmed as IS26‐ISKpn8‐blaKPC‐ΔISKpn6‐ΔtnpR‐Tn1721. Plasmids carrying *bla*_KPC_ variants were found to follow two distinct evolutionary pathways: one involving truncation of the Tn1721 transposon, retaining only the resolvase (tnpR) gene fragment and ISKpn27; the other involving replacement of ΔTnAs1 with IS26 or a bla_TEM‐1B_‐harboring ΔTn2 element. Notably, synonymous mutations were detected in the transposase (tnpA) and tnpR genes of some transposons carrying *bla*_KPC_ variants. Three promoters (designated PY, PX, and P1) were identified spanning the right inverted repeat (IRR) of ISKpn27 and the upstream non‐coding region of the bla_KPC_ variants. Additionally, some plasmids contained an extended promoter region including a bla_TEM‐1B_ gene fragment and a putative novel promoter (PZ), while others lacked either the PX or P1 promoter.

### 3.4 Virulence Factors of *K. pneumoniae*

Serotyping analysis confirmed that *K. pneumoniae* strains K2, K25, and K127 all belonged to capsular serotype K47. The ST11‐K47 *K. pneumoniae* lineage is known to harbor multiple virulence determinant gene clusters. Consistent with this, all three strains carried a large virulence plasmid (~190 kb) belonging to the IncHI1B(K)/FIB incompatibility group (Table 2). This virulence plasmid contained genes encoding heavy metal resistance determinants (copper and silver resistance) and key virulence factors, including the capsule polysaccharide regulator gene rmpA2 and the aerobactin iron acquisition system genes (iucABCD‐iutA). Sequence alignment showed that this ~190 kb virulence plasmid exhibits high homology (≥ 95%) to the previously reported pKP58‐1 plasmid (GenBank accession number: CP041374), which was first identified in clinical *K. pneumoniae* isolates from Hangzhou, China.

**Table 2:** Genetic characterization of strains K. pneumoniae K2, K. pneumoniae K25 and K. pneumoniae K127.

Strain	contig	Resistance and virulence genes	Inc type
K2	chromosome	aadA2 and bla_SHV‐182_	—
	pK2	bla_CTX‐M‐65_, bla_KPC‐2_, bla_SHV‐12_, and bla_TEM‐1B_	IncFII/IncR
K25	chromosome	aadA2 and bla_SHV‐182_	—
	pK25	bla_CTX‐M‐65_, *bla*_KPC‐25_, bla_SHV‐12_, and bla_TEM‐1B_	IncFII/IncR
K127	chromosome	aadA2 and bla_SHV‐182_	—
	pK127	bla_CTX‐M‐65_, bla_KPC‐127_, bla_SHV‐12_, and bla_TEM‐1B_	IncFII/IncR

### 3.5 Functional Characterization of KPC‐2, KPC‐25, and KPC‐127

To elucidate the molecular mechanisms underlying the altered susceptibility to ceftazidime‐avibactam and carbapenems mediated by KPC variants, recombinant KPC‐2, KPC‐25, and KPC‐127 proteins were purified to homogeneity for enzymatic kinetic assays. The catalytic efficiency (Kcat/Km) values demonstrated that KPC‐25 and KPC‐127 exhibited drastically reduced hydrolytic activity against meropenem and imipenem compared with wild‐type KPC‐2. This finding indicates that the diminished carbapenem hydrolytic activity of KPC‐25 and KPC‐127 contributes to the altered carbapenem susceptibility profiles observed in the corresponding clinical strains and transformants.

Relative to KPC‐2, both KPC‐25 and KPC‐127 showed decreased hydrolytic activity against ceftazidime. Importantly, the half‐maximal inhibitory concentration (IC_50_) values of avibactam for KPC‐25 and KPC‐127 were significantly higher than those for wild‐type KPC‐2, indicating reduced binding affinity of the KPC variants for avibactam. This reduced affinity for avibactam is a key factor contributing to the ceftazidime‐avibactam resistance phenotype. Integrating the antimicrobial susceptibility data of the transformants and the enzymatic kinetic results, although the hydrolytic activity of KPC‐25 and KPC‐127 against ceftazidime was decreased, the reduced affinity for avibactam (evidenced by higher IC_50_ values) allows the variant enzymes to retain sufficient catalytic activity against ceftazidime by escaping avibactam inhibition. These findings collectively explain the ceftazidime‐avibactam resistance phenotype mediated by KPC‐25 and KPC‐127 (Table 3).

**Table 3:** Kinetic parameters of the β‐lactamases KPC‐2, KPC‐25 and KPC‐127.

Antimicrobials	bla_KPC_ variants	*K*_*m*_ (μM)	*K*_cat_ (s^−1^)	*K*_cat_ */K*_*m*_ (μM^−1^s^−1^)	IC_50_
Imipenem	bla_KPC‐2_	312.6	163	0.52	/
	bla_KPC‐25_	720.5	4.86	0.0067	
	bla_KPC‐127_	653.8	7.41	0.011	
Meropenem	bla_KPC‐2_	106.12	100.21	0.94	/
	bla_KPC‐25_	64.53	6.32	0.098	
	bla_KPC‐127_	222.56	30.13	0.14	
Ceftazidime	bla_KPC‐2_	97.45	3.51	0.036	/
	bla_KPC‐25_	106.67	1.12	0.0104	
	bla_KPC‐127_	105.56	1.32	0.013	
Avibactam	bla_KPC‐2_	/	/	/	0.023
	bla_KPC‐25_				0.11
	bla_KPC‐127_				0.17

## 4 Discussion

Sequence type 11 (ST11) *bla*_KPC‐2_‐positive carbapenem‐resistant *Klebsiella pneumoniae* (CRKP) has become the dominant clonal lineage causing clinical infections in China, posing a severe challenge to anti‐infective therapy (Yang et al. 2022). Ceftazidime‐avibactam (CAZ‐AVI), a β‐lactam/β‐lactamase inhibitor combination with potent activity against KPC‐producing *K. pneumoniae*, was approved for clinical use in China in 2019 (Shirley 2018). However, its widespread application has been accompanied by the emergence of CAZ‐AVI‐resistant strains, primarily driven by mutations in the bla_KPC‐2_ gene (Ding et al. 2023). As demonstrated in our study, a 45‐year‐old patient with intracranial infection sequentially developed CAZ‐AVI resistance during treatment, with three ST11 K. pneumoniae strains (K2, K25, K127) isolated successively. These strains carried bla_KPC‐2_, bla_KPC‐25_, and bla_KPC‐127_, respectively, confirming the in vivo evolution of bla_KPC‐2_ to variant alleles under CAZ‐AVI selective pressure. This case is consistent with existing studies indicating that clinical use of CAZ‐AVI is a major risk factor for bla_KPC_ mutation, particularly in settings where sub‐inhibitory drug concentrations are present at the infection site (Shen et al. 2024).

Notably, the infection site in this case was the intracranial cavity, a unique anatomical location with a blood‐brain barrier that severely limits the penetration of antimicrobial agents. Previous studies have identified sub‐inhibitory concentrations of CAZ‐AVI at infection sites (e.g., lung, abdominal cavity) as a trigger for bla_KPC_ mutation in KPC‐producing *K. pneumoniae* (KPC‐KP) (Shen et al. 2024). For intracranial infections, achieving effective CAZ‐AVI concentrations is even more challenging due to the blood‐brain barrier, which may explain the rapid emergence of KPC‐25 and KPC‐127 variants in this patient. Winkler et al. demonstrated that increased avibactam concentrations can effectively inhibit KPC‐2 Ω‐loop variants and slow the development of CAZ‐AVI resistance (Winkler et al. 2015). Combined with our clinical observation—where the initial CAZ‐AVI regimen (2.5 g Q12h) failed to clear the infection and induced resistance—this suggests that the standard CAZ‐AVI dosage may be suboptimal for intracranial infections. We hypothesize that escalating the CAZ‐AVI dosage (e.g., 2.5 g Q6h) within a safe range, combined with therapeutic drug monitoring (TDM) to ensure adequate drug exposure at the intracranial site, could mitigate the risk of *bla*_KPC_ mutation and delay resistance development.

Molecularly, our study identified that bla_KPC‐25_ and bla_KPC‐127_ differ from bla_KPC‐2_ by mutations in the Ω‐loop region: bla_KPC‐25_ carries a 167_168dupLE insertion, while bla_KPC‐127_ harbors both 167_168dupLE and 172 A > T mutations. This aligns with global findings that over 60% of new KPC variants since 2019 arise from mutations in four loops surrounding the active site core (105 loop, Ω loop, 240 loop, 270 loop), with the Ω loop being the most common hotspot (Ding et al. 2023). The most well‐documented Ω‐loop mutation (D179Y) reduces avibactam inhibition while preserving ceftazidime hydrolysis, leading to variants such as KPC‐31 and KPC‐33 (the latter being prevalent in China) (Ding et al. 2023). Our enzymatic kinetic data further complement this mechanism: KPC‐25 and KPC‐127 exhibited decreased hydrolytic activity against ceftazidime but significantly higher avibactam IC_50_ values (reduced affinity) compared to KPC‐2. This allows the variant enzymes to escape avibactam inhibition and retain catalytic activity against ceftazidime, ultimately mediating CAZ‐AVI resistance. Notably, our study also found distinct carbapenem hydrolysis phenotypes between the two variants: KPC‐25 completely lost carbapenem hydrolytic activity, while KPC‐127 retained partial activity—an observation rarely reported in previous studies, highlighting the phenotypic diversity of Ω‐loop‐mutated KPC variants. Meanwhile, compared with KPC‐25, KPC‐127 carrying the 172 A > T substitution exhibits higher‐level resistance to ceftazidime‐avibactam. We hypothesize that this additional 172 A > T mutation counteracts the restored carbapenem susceptibility conferred by the 167_168dupLE insertion and further elevates the resistance toward ceftazidime‐avibactam.

The genetic environment of bla_KPC_ genes in this study—IS26‐ISKpn8‐bla_KPC_‐ΔISKpn6‐ΔtnpR‐Tn1721—provides new insights into the dissemination mechanism of bla_KPC_ variants in China. Globally, the bla_KPC_ gene is most commonly carried by the Tn4401 transposon, while Chinese isolates frequently harbor Tn3‐Tn4401 hybrid transposons (Huang et al. 2020). In contrast, our strains carried bla_KPC_ alleles within an IS26‐truncated Tn1721 transposon, suggesting that Tn1721 chimeras may represent a novel non‐Tn4401 mobile element evolved via genetic recombination. All genomic deletions/insertions in our KPC‐harboring strains were associated with IS26, which can form composite transposons via two tandem copies to facilitate transposition. This further confirms that IS*26*‐mediated horizontal gene transfer plays a crucial role in the emergence and diversification of bla_KPC_ variants. Additionally, whole‐genome sequencing revealed that all three strains carried an ~190 kb IncHI1B(K)/FIB‐type virulence plasmid (homologous to pKP58‐1, GenBank: CP041374) encoding heavy metal resistance (copper, silver) and key virulence factors (rmpA2, iucABCD‐iutA). This is consistent with the ST11‐K47 lineage's characteristic of harboring multiple virulence determinant clusters, indicating that the coexistence of drug resistance and virulence genes in these strains may enhance their adaptability in clinical settings and worsen patient prognosis.

The emergence of such KPC variants poses significant challenges to laboratory diagnostics and clinical management, which are particularly relevant to our case:

1. Risk of false‐negative laboratory detection: Conventional carbapenemase detection methods (e.g., CLSI‐recommended mCIM/eCIM, carbapenemase inhibitor‐enhanced tests, immunochromatographic assays) may yield false negatives for KPC variants. This is likely due to conformational changes in mutant KPC proteins (e.g., Ω‐loop mutations in KPC‐25 and KPC‐127) that reduce binding efficiency with monoclonal antibodies in immunochromatographic assays, leading to detection failure. Given that our strains were isolated from intracranial infection (a life‐threatening condition), false‐negative results could delay targeted therapy and increase mortality.
2. KPC variants are often inhibited by clavulanic acid, which may cause automated susceptibility testing systems to misclassify them as ESBL‐producing organisms. This atypical profile could lead to inappropriate empirical therapy—for example, selecting ESBL‐active agents instead of carbapenems for KPC‐25‐positive strains, or continuing CAZ‐AVI for CAZ‐AVI‐resistant variants. Notably, our study further indicates that even though mutant strains (e.g., KPC‐25‐positive strain) regain susceptibility to carbapenems, carbapenem monotherapy should not be adopted. This is because carbapenem exposure can rapidly induce the re‐emergence of carbapenem resistance in these strains, ultimately resulting in treatment failure.
3. Lack of standardized treatment regimens: Currently, there are no globally standardized treatment guidelines for KPC variant‐associated infections. In our case, salvage therapy with CAZ‐AVI (2.5 g q8h) plus aztreonam achieved clinical cure, which provides a potential therapeutic option for such infections.

These challenges suggest that the true prevalence of KPC variant strains may be underestimated in clinical settings, particularly in severe infections such as intracranial infections where diagnostic delays are common. To address this, establishing a global surveillance network focused on KPC variants is imperative. This network should monitor clinical detection rates, infection risk factors (e.g., CAZ‐AVI exposure, severe infection types), diagnostic performance of existing methods, and therapeutic outcomes. Additionally, optimizing diagnostic strategies—such as integrating whole‐genome sequencing into routine diagnostics for severe CRKP infections—could improve the detection of KPC variants and reduce false negatives. For clinical practice, TDM‐guided CAZ‐AVI dosing, especially in patients with severe infections or impaired drug penetration (e.g., intracranial, abscess), may help reduce the emergence of KPC variants.

In conclusion, our study reports the sequential emergence of KPC‐25 and KPC‐127 variants in an intracranial infection patient during CAZ‐AVI treatment, are consistent with that the role of sub‐inhibitory drug concentrations (exacerbated by the blood‐brain barrier) in driving bla_KPC_ mutation. The unique genetic environment (IS*26*‐truncated Tn*1721*) and coexistence of virulence genes highlight the complex evolution of ST11 *K. pneumoniae*. The clinical challenges posed by KPC variants emphasize the need for enhanced surveillance, improved diagnostic methods, and personalized therapeutic strategies to combat this emerging threat.

## Author Contributions

**Ke Lei:** writing – review and editing, writing – original draft, resources, software, investigation, data curation, conceptualization. **Ying Tian:** formal analysis, supervision, validation. **Chaoliang Xiong:** investigation, methodology. **Jing Lei:** methodology, software. **Dong Chen:** methodology, software. **Xiangni Bai:** methodology, software. **Mohd H. Abdul‐Aziz:** formal analysis, visualization, project administration. **Jiao Xie:** formal analysis, supervision, funding acquisition. **Zeshi Liu:** supervision, funding acquisition, visualization, project administration, resources.

## Ethics Statement

All experiments conducted in this study were performed in strict accordance with relevant laws, regulations, and institutional guidelines. No human or animal subjects were involved in this research. The collection and use of bacterial isolates were carried out following standard microbiological protocols and approved biosafety procedures. The ethics committee waived the requirement for informed consent from the participants, ethics approval number: 2025(034).

## Consent

The authors have nothing to report.

## Conflicts of Interest

The authors declare no conflicts of interest.

## Data Availability

The genomic information of *K. pneumoniae* PTL has been deposited at NCBI (National Center for Biotechnology Information) under the accession number PRJNA1401311, PRJNA1401312, and PRJNA1401316.
